# Adiponectin gene polymorphisms and risk of type 2 diabetes: an updated evidence for meta-analysis

**DOI:** 10.1186/s13098-021-00749-x

**Published:** 2021-11-17

**Authors:** Mahrokh Alimi, Mohammad Taghi Goodarzi, Mehdi Nekoei

**Affiliations:** 1 Department of Chemistry, Shahrood Branch, Islamic Azad University, Shahrood, Iran; 2 Department of Biochemistry, Shahrood Branch, Islamic Azad University, Shahrood, Iran

**Keywords:** Adiponectin, Type 2 diabetes, SNP − 11377, SNP + 276, Single nucleotide polymorphism

## Abstract

**Background:**

Growing body of evidence suggest the association between SNP − 11377 C > G and SNP + 276 G > T polymorphisms of adiponectin gene with type 2 diabetes (T2D). However, these findings have not been conclusive and consistent. The present study quantitatively evaluates the data on the association between DIPOQ − 11377C/G, and + 276G/T polymorphisms and risk of T2D through a meta-analysis.

**Methods:**

A systematic search was performed in the PubMed, Web of science, Scopus and Cochrane library databases to extract published studies according to the inclusion criteria. Among the 741 studies, 391 of them were screened as full text and 31 studies were finally included in the meta-analysis. Analysis of data was performed using random-effects model. Odds ratios (ORs) with 95% confidence intervals (CIs) were used to analyze the strength of association. Subgroup and meta-regression analyses were performed to identify the potential source of heterogeneity.

**Results:**

The pooled analysis showed that there was no statistically significant association between genotypes of CC (OR = 0.76, 95% CI: 0.53–1.09, *P* = 0.14), CG (OR = 0.93, 95% CI: 0.72–1.20, P = 0.58) and GG (OR = 1, 95% CI: 0.80–1.26, P = 0.94) ADIPO − 11377 polymorphism with increased risk of T2D. In addition, the results revealed a trend toward an increased risk of T2D for the SNP + 276 TT genotype (OR = 0.87, 95% CI: 0.77–0.98, P = 0.026) as compared with the GT and GG genotypes. Subgroup analysis by ethnicity indicated significant association between the TT genotype of the SNP + 276 and increased risk of T2D among Europeans. Met-regression demonstrated significant association between the GT genotype of + 276 polymorphism with risk of T2D in male individuals (slope: 0.0006; 95% CI: 0.0002–0.0009; P < 0.001).

**Conclusions:**

Collectively, our findings demonstrated a positive association between ADIPOQ + 276 G > T polymorphism with increased risk of T2D in male individuals with European ethnicity.

**Graphical Abstract:**

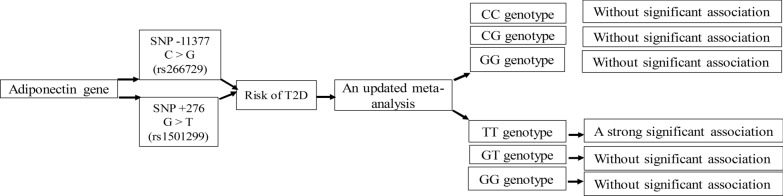

## Highlights

Adiponectin (ADIPOQ) plays an important role in the development of type 2 diabetes and previous studies regarding the association between ADIPOQ polymorphisms and T2D risk reported conflicting results.

We performed a meta-analysis to assess the association between two ADIPOQ polymorphisms [− 11377C/G (rs266729), and +276G/T (rs1501299)] and risk for T2D.

Odds ratios (ORs) with corresponding 95% confidence intervals (95% CIs) were pooled to assess the association between four aforementioned polymorphisms and susceptibility to T2D.

Overall, there was an association between ADIPOQ +276 G > T with increased T2D risk.

Subgroup by ethnicity suggested significant association between +276G/T polymorphism and T2D risk among European ethnicities.

Our findings evidence a positive association between ADIPOQ +276 G > T polymorphism with increased risk of T2D in male individuals with European ethnicity.

## Background

Type 2 diabetes (T2D), a metabolic disorder with severe complications, is a growing public health problem all over the world [1]. According to the latest statistics published by the International Diabetes Federation (IDF), about 500 million people worldwide have confirmed or diagnosed diabetes. It’s predicted that approximately 693 million people will be affected by the disease by 2045[2]. Although insulin resistance is the main property of patients with T2D, some grade of insulin resistance has been observed in nondiabetic individuals [3]. It has been suggested that insulin resistance in T2D is caused by a combination of genetic and environmental factors [4]. Environmental risk factors contributing to T2D include central obesity, low exercise, high fat nutrition, hypertension, and impaired glucose tolerance [5, 6]. Furthermore, genetic factors play a potential role in the development of the disease among different populations. Individuals with genetic susceptibility have a higher risk of developing T2D than other people [4]. Several polymorphisms have been reported to be associated with T2D risk. Accumulating reports have revealed the main role of adiponectin gene polymorphism in the development of T2D [7]. The circulating levels of adiponectin, an adipose tissue-extracted protein (~ 30 kDa), have been shown to be decreased in patients with metabolic syndrome including T2D, and insulin resistance [8]. It is known that adiponectin has anti-inflammatory, anti-atherosclerotic, and antidiabetic properties [9]. Furthermore, it has been reported that plasma levels of adiponectin are also decreased in T2D condition. The ADIPOQ, adiponectin-coding gene, which is located in chromosome 3q27, proposed as a genomic locus for the T2D using genome-wide scans [7, 8]. The association of two common single nucleotide polymorphisms (SNPs) of ADIPOQ gene, rs266729 and rs1501299 with risk of T2D have been investigated in different populations worldwide [10, 11]. Given the many contradictions in the effect of adiponectin gene polymorphism in rs266729 and rs1501299 locus on T2D, we designed a study to find out the association of ADIPOQ gene polymorphism, rs266729 and rs1501299, with risk of T2D using meta-analysis.

## Methods

### Search strategy

This study was outlined according to the guidelines of the 2009 Preferred Reporting Items for Systematic Reviews and Meta-Analysis (PRISMA) statement [12]. To find the full texts related to this topic, our search was performed on PubMed, Scopus, Web of Science, and Embase databases up to July 15, 2021. The above databases were searched based on the following queries as key terms in the title and abstract: (Adiponectin gene polymorphism OR single nucleotide polymorphism (SNP) − 11377 C > G OR SNP − 11365 C > G AND type 2 diabetes), (Adiponectin gene polymorphism OR SNP + 276 G > T AND type 2 diabetes), (SNP + 276 OR rs1501299 of adiponectin gene AND type 2 diabetes), (SNP − 11377 OR rs266729 of adiponectin gene AND type 2 diabetes). The wild-card term "*" was used to improve the sensitivity of the search strategy. We enhanced this search by scanning the reference lists of relevant articles. The search was limited to previous studies published in English language. Two researches (MA and MT. G) assessed the full text independently, Disagreements were resolved by consensus-based discussion by third party (M. N).

### Inclusion and exclusion criteria

According to the topic of this study, inclusion criteria included: (1) observational (cohort, case –control) studies which evaluated the association between the polymorphisms of SNP − 11377 and SNP + 276 of adiponectin gene in patients with T2D and healthy controls, (2) studies providing relevant information with topic such as genotype frequency for assessment of odds ratio (ORs) and 95% confidence intervals (95% CI), (3) studies with human subjects, (4) the control group with healthy individuals. The following items were excluded from the study: (1) animal and in vitro studies, (2) observational studies were not related to the association between the polymorphism of adiponectin gene and T2D, (3) conference abstracts, reviews, case reports, or editorials; (4) lack of sufficient information on adiponectin gene polymorphisms and susceptibility to T2D, and (5) studies with insufficient details of study methodology.

### Study selection

To find the original articles associated with this subject, titles and abstracts of all retrieved studies were screened separately by two reviewers to identify the relevant articles. According to the predefined inclusion and exclusion criteria, the articles were selected for the meta-analysis.

### Data extraction

Selected articles were reviewed and data were independently retrieved by two researches using a standardized electronic form. The following information was extracted: (1) first author name, (2) publication year, (3) study location (country), (4) study designs which were classified into case–control and cohort, (5) genotyping methods, (6) ethnicity, (7) target population which was categorized as Asian, American, African and European, (8) sample sizes of case and controls (cases are individuals with T2D), (9) genotype and/or allele frequencies in case and control groups, (10) P-value of Hardy–Weinberg equilibrium (HWE) and (11) Newcastle Ottawa Scale (NOS) score. Above data were extracted for two polymorphisms of SNP − 11377 and SNP + 276 separately.

### Quality assessment

The quality of all eligible studies was assessed by two researches using the Newcastle Ottawa Scale (NOS) [13]. The NOS is composed of 3 items: selection, comparability, and exposure, with a total score of 9. Based on the final score, the studies could be classified into high quality (score more than 6), medium quality (score between 4 and 6), and low quality (score less than 4). Any disagreements were adjudicated through discussion.

### Statistical analysis

The data were analyzed based on random-effect model. Effect size was expressed as odds ratio with 95% CIs to evaluate the associations between SNP − 11377 C > G (rs266729) and SNP + 276 G > T (rs1501299) of adiponectin gene and risk of T2D. Higgin’s index and Cochrane's Q test were applied to evaluate heterogeneity among included studies. If the Higgin's index revealed a P-value of I^2^ < 50%, the fixed-effects model (the Mantel–Haenszel method) was selected to pool the data. Otherwise, the random effects model (the DerSimonian and Laird method) was used. Potential publication bias was calculated using Begg's funnel plot, the funnel plot of the study precision (inverse standard error) by effect size (log OR) and Egger’s weighted regression tests [14, 15]. If publication bias observed, Duval and Tweedie "trim and fill" method was used to adjust pooled OR and 95% CI. Sensitivity analysis was performed using “leave-one-out” method with removing one study in turn and reporting the analysis. Subgroup and random-effect meta-regression were conducted. Subgroup analyses were performed based on subgroups of study design (cohort and case–control), ethnicity (Asian, American, European and African) and sex (male and female). To find significant heterogeneity, meta-regression was performed using unrestricted maximum likelihood method to assess the association between estimated effect size of the polymorphisms of adiponectin gene and risk of T2D with variables of sex, age and ethnicity. Statistical analyses were conducted using the Comprehensive the Comprehensive Meta-Analysis (CMA) V2 software (Biostat, NJ) [16].

## Results

### Process of study selection

**Fig. 1:**
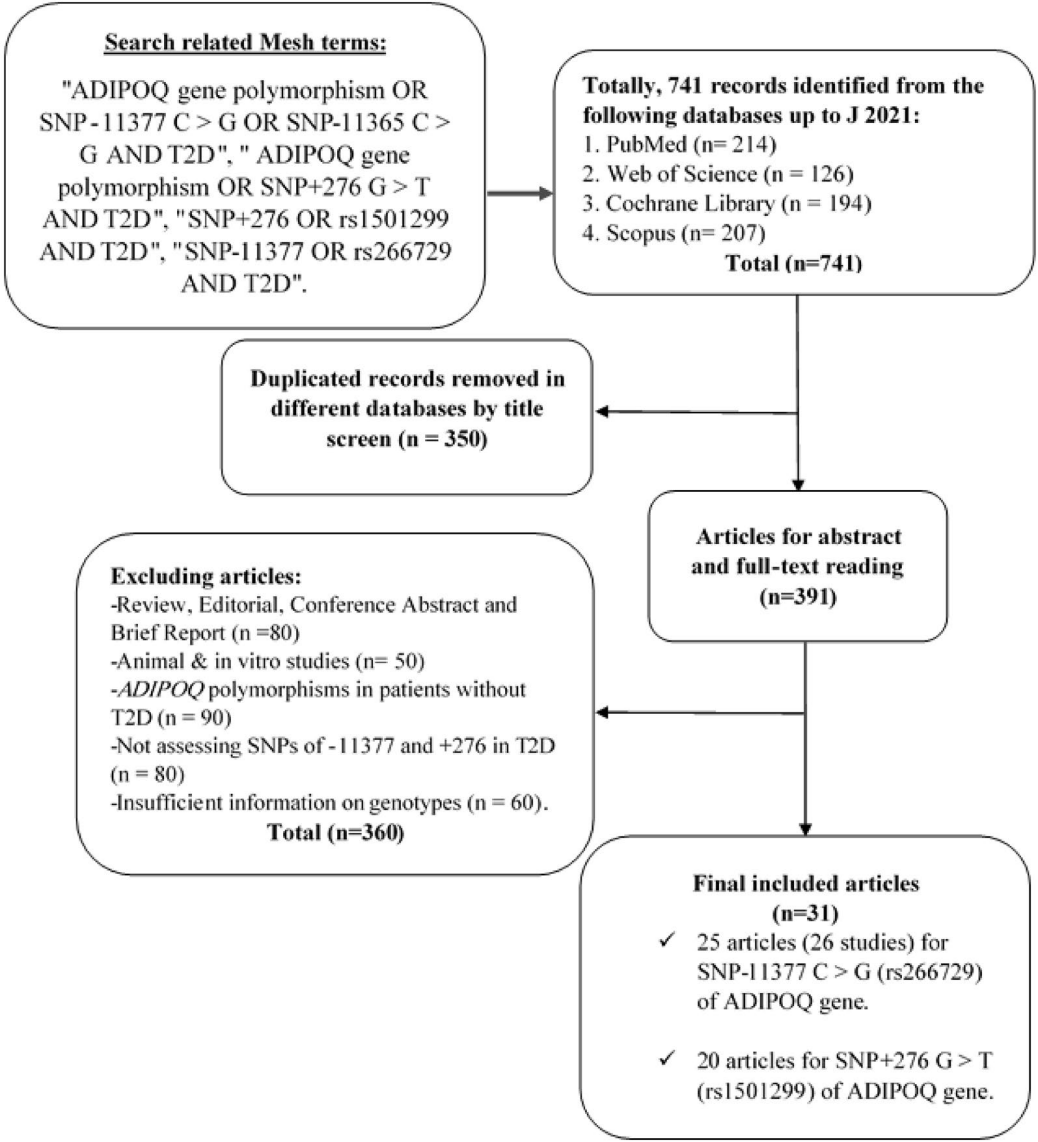
Search flow diagram of study selection

The search provided 741 records. 350 items were removed from the study because of the duplication. After screening of the title and abstract of full text, 31 studies were identified according to the inclusion criteria. 25 relevant studies were included in − 11,377 C > G (rs266729) analysis and 20 articles were included in + 276 G > T (rs1501299) analysis. Some articles included in this meta-analysis was common for these SNPs. The reasons for excluding the remaining 360 articles were: articles that were reviews, editorial, conference abstract and brief report (n = 80), articles that were performed in animal and in vitro models (n = 50), articles evaluating adiponectin gene polymorphisms in patients without T2D (n = 90), articles didn’t assess SNPs of − 11377 and + 276 in T2D patients (n = 80), articles didn’t provide sufficient information on the numbers of genotypes (n = 60). A summary of the study selection process according to the PRISMA flowchart is shown in Fig. 1.

### Characteristics of included studies

In total, 31 studies were included according to the inclusion criteria. Out of these 31 studies, 25 studies were allocated to evaluation of SNP − 11377 C > G (rs266729) in subjects with T2D and 20 studies to SNP + 276 G > T (rs1501299) analysis. Of 20 studies for the analysis of SNP + 276, only 6 studies were different. In the 25 studies associated with − 11377 C > G, 11,963 cases and 15,527 controls were included. Of 11,963 cases, 6098 cases were males and 5865 cases were females. In the 20 studies involved in + 276 G > T analysis, 8658 cases and 16,498 controls were included. In the cases group of SNP + 276 analysis, 4130 cases were males and 4528 cases were females. Of the 25 studies for analysis of SNP − 11377 C > G, 18 were case–control studies and seven were cohort studies. Among 20 studies for SNP + 276 G > T analysis, 13 studies were case–control studies, six studies were cohort studies and one study was a cross-sectional. Selected studies were conducted in individuals with T2D. Included articles were published between 2002 and 2021. The examined races were Asian, American, European and African. The baseline and demographic characteristic of the eligible studies were summarized in Tables 1 and 2.

**Table 1:** Main characteristics of studies included for investigation of association between SNP − 11377 (rs266729) and risk of type 2 diabetes

Author’s name	Country	Study design		Sex ratio (M/F)	Genotyping method		Ethnicity	Target population	Disease	Sample size (Diabetes)	Sample size (control)	Case			Control			P-value for HWE	NOS score
												GG	CG	CC	GG	CG	CC		
Zhang et al. [17]	Sweden	Cohort		313/265	DASH		Caucasian	American	T1D	578	599	35	232	310	29	230	340	0.022	7
Hara et al. [18]	Japan	Case–control		267/117	SNaPshot ddNTP		Asian	Japanese	T2D	384	480	24	127	233	37	178	265	0.77	8
Vasseur et al. [19]	France	Case–control		604/769	Ampli-Fluor		Caucasians	French	T2D	1373	743	46	274	300	45	264	382	0.71	8
Gu et al. [20]	Sweden	Case–control		56/50	DASH		Caucasians	Swedish	T2D	106	497	11	34	58	53	186	247	0.25	8
Hu et al. [21]	USA	Nested Case–control		642F	TaqMan		Caucasians	Caucasians	T2D	642	995	41	244	357	59	379	557	0.75	9
Gibson et al. [22]	UK	Case–control		390/422	Ampli-Fluor		Caucasians	French	T2D	812	1044	59	320	433	70	402	572	0.003	7
Vasseur et al. [23]	France	Case–control		115/329	TaqMan		Caucasians	French	T2D	444	535	10	98	123	13	90	167	0.04	8
Tso et al. [24]	Hong Kong	Cohort		76/82	PCR–RFLP		Chinese	Chinese	T2D	50	104	10	62	86	8	44	52	0.1	7
Schwarz et al. [25]	Germany	Cohort		224/191	TaqMan		Caucasians	German	T2D	365	323	35	143	187	20	120	183	0.003	7
Gable et al. [26]	UK	Cohort		169 M	TaqMan		Caucasians	European	T2D	169	2767	15	60	83	175	1038	1440	0.43	6
Olckers et al. [27]	South Africa	Cohort		31/196	TaqMan		Black	South African	T2D	227	226	1	60	166	5	60	161	0.05	6
Sun et al. [28]	China	Case–control		138/117	PCR–RFLP		Chinese	Chinese	T2D	255	120	14	119	122	5	41	74	0.05	7
Hivert et al. [29]	USA	Cohort		515/580	MS		white	white	T2D	1095	1448	11	98	124	146	784	1259	2.52	7
Wang et al. [30]	China	Case–control		394/591	TaqMan		Han Chinese	Han Chinese	T2D	985	1050	79	379	479	61	408	529	2.31	7
Yang et al. [31]	China	Case–control		75/137	PCR–RFLP		Han Chinese	Han Chinese	T2D	212	585	22	90	100	50	210	325	3.6	7
Wang et al. [32]	China	Case–control		196/142	PCR–RFLP		Chinese	Chinese	T2D	338	460	21	101	165	15	161	243	3.53	8
Chiodini et al. [33]	Italy	Cohort		285/218	TaqMan		Italians	Italians	T2D	503	503	22	159	322	22	160	321	0.72	9
Karimi et al. [34]	Iran	Case–control		36/44	PCR–RFLP		Iranian	Iranian	T2D	80	80	2	32	441	1	29	50	0.65	6
Palit et al. [35]	India	Case–control		142/143	PCR–RFLP		Gujarat	Indian	T2D	285	286	148	148	137	131	131	155	0.2	7
Palit et al.	India	Case–control		217/174	PCR–RFLP		Jand K	Indian	T2D	503	290	194	194	309	139	139	151	0.2	8
Ramya et al. [36]	India	Case–control		497/603	PCR–RFLP		South Indian	Indian	T2D	1073	1053	71	71	572	49	383	621	0.004	6
Mtiraoui et al. [37]	Tunisia	Case–control		422/495	TaqMan		Caucasian	Tunisia	T2D	917	748	89	89	4231	50	279	419	5.1	9
Cui et al. [4]	China	Case–control		85/118	IMLDR		Chinese	Chinese	T2D	203	203	10	10	110	15	83	105	0.47	7
Nomani et al. [38]	Iran	Case–control		43/57	PCR–RFLP		Iranian	Iranian	T2D	100	95	3	3	61	1	28	66	0.35	8
Alimi et al. [11]	Iran	Case–control		57/43	Tetra ARMS-PCR		Iranian	Iranian	T2D	100	110	9	9	33	9	46	55	0.041	8
Populaire et al. [39]	Japan	Case–control		82/82	PCR–RFLP			Japanese	T2D	164	183	11	11	105	10	87	86	0.002	6

**Table 2:** The baseline information of studies included for assessment of association between SNP + 276 (rs1501299) and risk of type 2 diabetes

Author's name	Country	Study design	Sex ratio of case (M/F)	Genotyping	method	Ethnicity	Target population		Sample size (Diabetes)	Sample size (Control)	Case			Control			P-value for HWE	NOS score
											TT	GT	GG	TT	GT	GG		
Hara et al. [18]	Japan	Case–control	267/117	SNaPshot ddNTP		Asian	Japanese		384	480	18	142	224	41	203	236	0.08	8
Menzaghi et al. [40]	Italy	Case–control	167/143	PCR-hydridization		Caucasian	Italian Caucasian		310	304	28	124	158	27	117	160	0.7	8
Populaire et al. [39]	Japan	Case–control	82/82	PCR–RFLP		Asian	Japanese		164	177	22	55	87	18	80	79	0.12	9
Gu et al. [20]	Sweden	Case–control	56/50	DASH		Caucasian	Swedish Caucasians		106	497	101	46	50	42	206	249	0.004	8
Fumeron et al. [41]	France	Cohort	150/79	fluorogenic		Caucasian	French Caucasian		229	3072	15	64	97	305	1773	2419	0.67	7
Hu et al. [21]	USA	Nested Case–control	642F	TaqMan		Caucasian	Caucasians		642	995	54	266	322	73	399	523	0.07	9
Gibson et al. [22]	UK	Case–control	390/422	Ampli-Fluor		Caucasian	French Caucasian		701	893	51	276	374	75	368	450	0.0003	7
de Courten et al. [42]	USA	Cohort	496/584	PCR–RFLP		Indian	Pima Indians		1080	1080	26	200	371	24	133	249	1.2	7
Lee et al. [43]	Korea	Case–control	237/256	SNaPshot ddNTP		Korean	Korean population		493	427	38	231	224	35	167	225	0.26	6
Sanchez et al. [44]	Finland	Cross-sectional	356/391	PCR-SnaPshot		Spanish	Spanish		747	747	5	35	24	39	231	260	1.61	6
Vasseur et al. [23]	France	Case–control	115/329	LightCycler technology		Caucasians	French Caucasians		444	535	23	89	97	23	93	132	0.34	8
Tso et al. [24]	Hong Kong	Cohort	76/82	PCR–RFLP		Chinese	Chinese		137	125	17	59	82	15	40	49	2	7
Gable et al. [26]	UK	Cohort	169 M	TaqMan		Caucasians	European Caucasian		169	2767	10	59	87	175	1015	1470	0.92	6
Yang et al. [45]	China	Case–control	75/137	SEQUENOM genotyping system		Chinese	Chinese		637	801	42	206	191	661	408	499	2.04	8
Hivert et al. [29]	USA	Cohort	515/580	Mass spectroscopy		White	White		282	1448	15	61	100	111	641	864	0.28	7
Magdalena et al. [46]	Poland	Case–control	217/278	PCR–RFLP		Caucasian	Polish Caucasian		495	435	11	131	353	11	150	274	0.024	9
Mohammadzadeh et al. [47]	Iran	Case–control	26/24	PCR–RFLP		Iranian	Iranian		50	52	2	19	29	4	21	27	0.41	8
Wang et al. [32]	China	Case–control	394/591	TaqMan		Han	Han Chinese		985	1050	66	397	451	72	398	496	0.41	7
Chiodini et al. [33]	Italy	Cohort	285/218	TaqMan		Italians	Italians		503	503	52	206	245	66	198	239	0.02	9
Alimi et al. [11]	Iran	Case–control	57/43	Tetra ARMS-PCR		Iranian	Iranian		100	110	8	45	47	8	44	58	0.3	6

### Quality assessment

Evidence quality scores were summarized in Tables 1 and 2 as a column. According to the NOS score, quality scores ranged from 7 to 9. The seven demonstrates a moderate score and 9 means a strong score. The quality of the included studies was moderate to strong in all studies.

## Meta-analysis results

### Association between the SNP − 11337 C > G polymorphism of adiponectin gene and risk of T2D

**Fig. 2:**
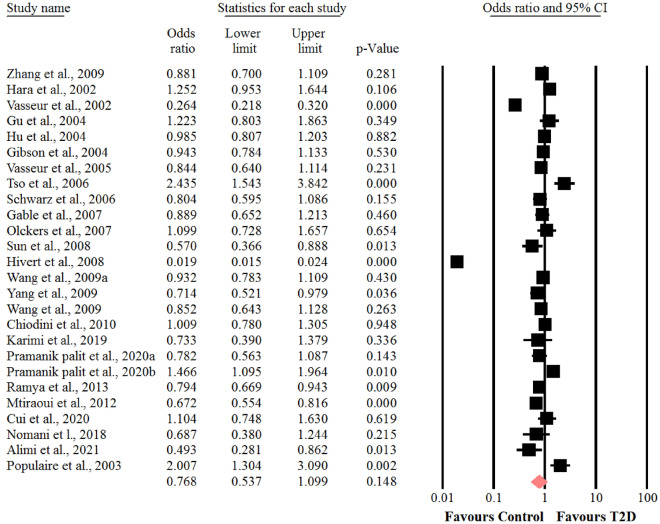
Forest plot for the association between the GG genotype of SNP  − 11377 C > G and risk of T2D in the whole populations

**Fig. 3:**
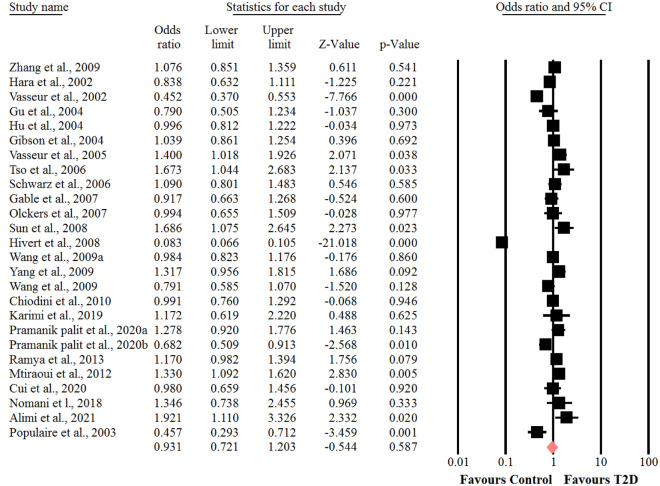
Forest plot for the association between the CG genotype of SNP  − 11377 C > G and risk of T2D in the whole populations

**Fig. 4:**
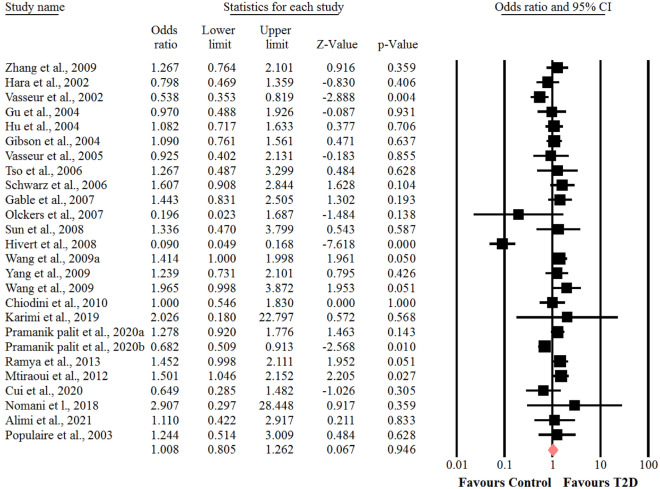
Forest plot for the association between the GG genotype of SNP  − 11377 C > G and risk of T2D in the whole populations

Meta-analysis of data showed a significant heterogeneity among the included studies of genotyping frequencies of CC (I^2^ = 97.7%, *P* < 0.001), CG (I^2^ = 95.3%, *P* < 0.001) and GG (I^2^ = 75.7%, *P* < 0.001) of − 11377 C > G polymorphism of adiponectin gene and risk of T2D. Thus, the random-effects model was used. The results revealed that there were no significant association between genotypes of CC (OR = 0.76, 95% CI: 0.53–1.09, *P* = 0.14) (Fig. 2), CG (OR = 0.93, 95% CI: 0.72–1.20, *P* = 0.58) **(**Fig. 3) and GG (OR = 1, 95% CI: 0.80–1.26, *P* = 0.94) (Fig. 4) of − 11377 C > G polymorphism and risk of T2D. In addition, we performed sensitivity analysis using “leave-one-out” method to assess the stability of overall results. The effect sizes obtained from three genotypes were robust and removing any of the studies in turn did not change the effect on estimated overall results significantly.

### Association between the SNP + 276 G > T polymorphism of adiponectin gene and risk of T2D

**Fig. 5:**
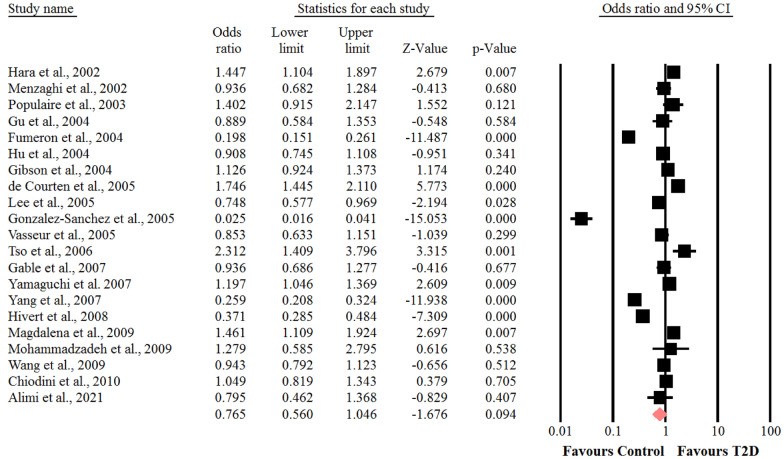
Forest plot for meta-analysis correlation between the TT genotype of SNP + 276 G > T (rs1501299) and T2D risk in the overall populations

**Fig. 6:**
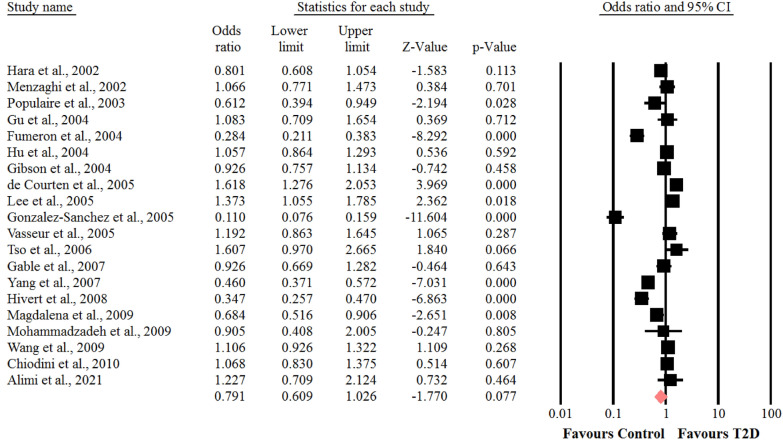
Forest plot for meta-analysis correlation between the GG genotype of SNP + 276 G > T (rs1501299) and T2D risk in the overall populations

**Fig. 7:**
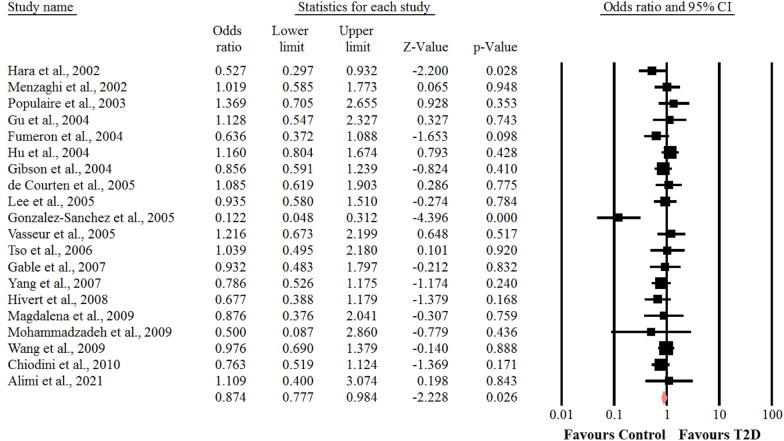
Forest plot for meta-analysis correlation between the GT genotype of SNP + 276 G > T (rs1501299) and T2D risk in the overall populations

With respect to the presence of obvious heterogeneity among the genotypes of GT (I^2^ = 93.8%, *P* < 0.001) and GG (I^2^ = 96.7%, *P* < 0.001). The random-effects model was adopted, whereas for the analysis of the genotype of TT (I^2^ = 38%, *P* = 0.05), the fixed- effects model was used. In the whole populations including SNP + 276 G > T polymorphism, a significant association was found between the genotype TT of SNP + 276 G > T polymorphism of adiponectin gene and susceptibility to T2D (OR = 0.87, 95% CI: 0.77–0.98, *P* = 0.026) (Fig. 5). There was no statistical significant association between the genotypes of GT and GG of SNP + 276 G > T polymorphism of adiponectin gene and risk of T2D (OR = 0.79, 95% CI: 0.60–1.02, *P* = 0.07) (Fig. 6) and (OR = 0.76, 95% CI: 0.56–1.04, *P* = 0.09) (Fig. 7), respectively. We performed sensitivity analysis using “leave-one-out” method by removing the studies by sequence. The results showed that omission of the studies did not have a significant effect on pooled ORs. These results indicating that our meta-analysis had reliable and stable results.

### Publication bias

**Fig. 8:**
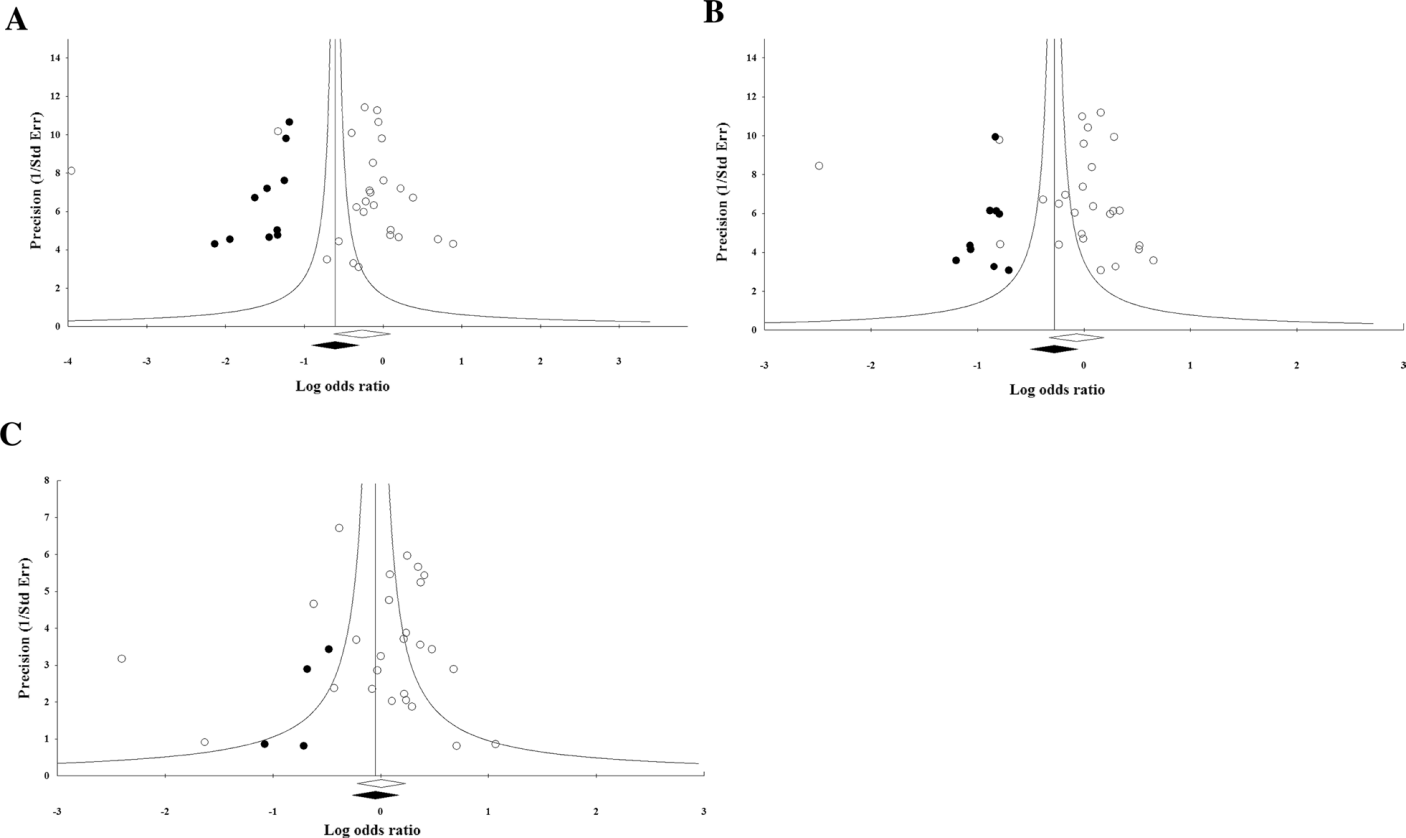
Funnel plots for assessing publication bias in the studies investigating association between SNP − 11377 C > G (rs266729) and risk of T2D in the genotype models: **A** CC, **B** CG, **C** GG

**Fig. 9:**
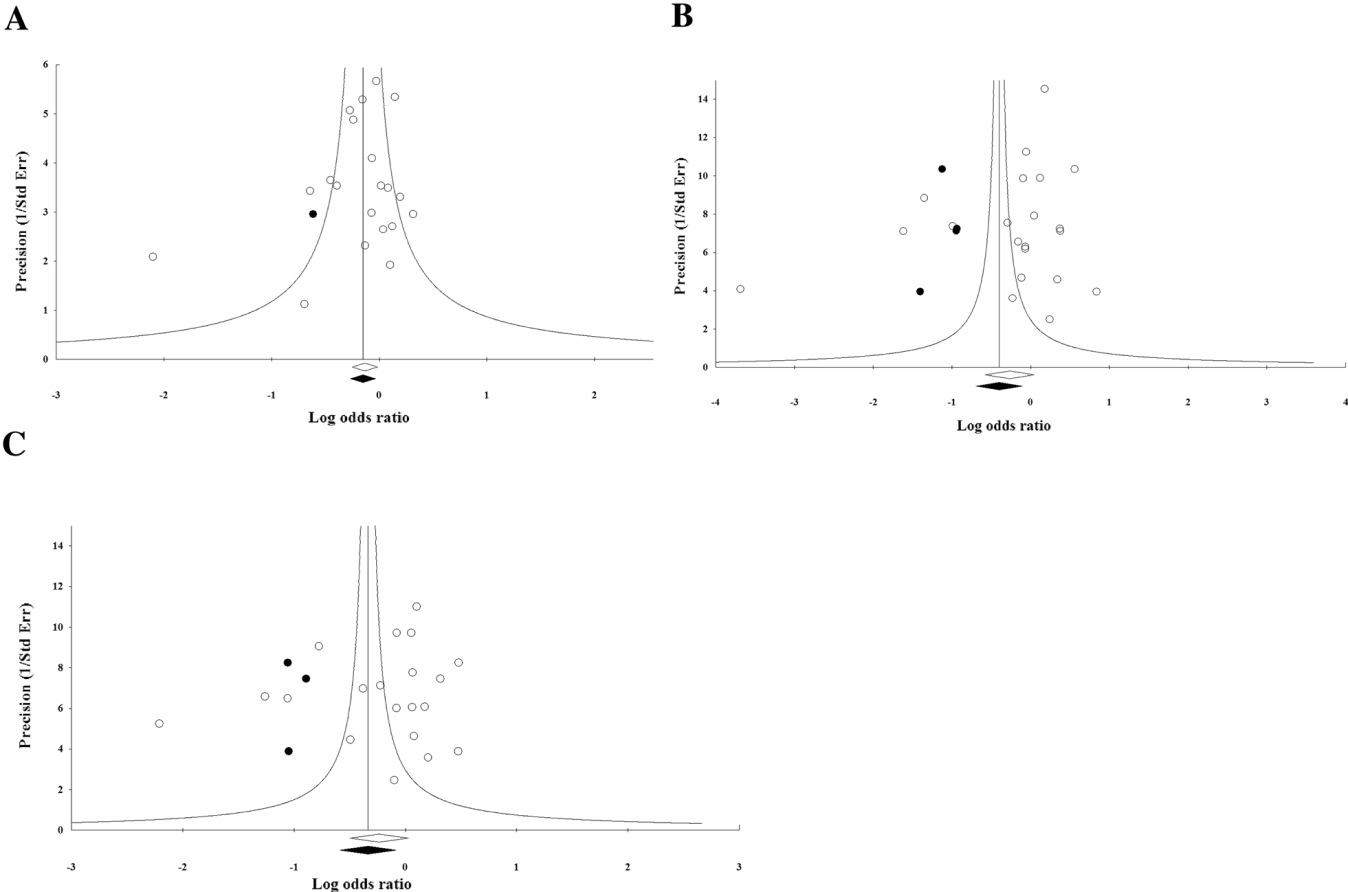
Funnel plots for assessing publication bias in the studies investigating association between SNP + 276 G > T (rs266729) and risk of T2D in the genotype models: **A** TT, **B** GG, **C** GT

Funnel plot, Begg's rank correlation and Egger’s regression tests were performed to find the potential publication bias among the studies. Visual inspection of the funnel plots of genotypes in polymorphisms of − 11377 and + 276 showed no significant publication bias among included studies (Fig. 8). Begg’s rank correlation test for genotype of CC (P = 0.62), CG (P = 1) and GG (P = 0.42) of − 11377 C > G polymorphism and for genotypes of TT (P = 0.58), CG (P = 0.29) and GT (P = 0.25) of + 276 G > T polymorphism were not significant. In addition, the results of Egger’s regression test also showed no evidence of significant publication bias for genotypes of − 11377 polymorphism (P = 0.49 for CC, P = 0.51 for CG and P = 0.80 for GG) and for genotypes of + 276 polymorphism (P = 0.30 for TT, P = 0.27 for GG and P = 0.49 for GT). The observed publication bias was imputed using trim-and-fill method. 23 missing studies for the genotypes of CC, CG and GG of − 11377 polymorphism were imputed leading to correct pooled analysis that less than the overall effect size (OR: 0.54; 95% CI: 0.40–0.73), (OR: 0.75; 95% CI: 0.60–0.94) and (OR: 0.95; 95% CI: 0.76–1.17), respectively. The classic fail-safe N method showed that collectively 898 theoretically missing studies for − 11377 polymorphism would be needed to determine the pooled calculated effect size significantly. In addition, eight potentially missing studies were imputed for the genotypes of TT, GT and GG of + 276 polymorphism that equal with the initial estimate (OR: 0.84; 95% CI: 0.72–0.98), (OR: 0.54; 95% CI: 0.40–0.73) and (OR: 0.54; 95% CI: 0.40–0.73), respectively. The classic fail-safe N method for + 276 polymorphism indicated that 457 theoretically missing studies were required to bring P-value to < 0.05 (Figs. 8, 9).

### Subgroup analyses

Subgroup analyses were conducted to find the possible source of significant heterogeneity among the included studies in − 11377 C > G and + 276 G > T polymorphisms of adiponectin gene using the following subgroups: sex (male *vs*. female), study design (case–control *vs*. cohort) and ethnicity (American vs. European vs. Asian vs. African).

### Subgroup analysis of − 11377 polymorphism

The results of subgroup analyses for genotypes of CC, CG and GG of − 11377 C > G polymorphism of adiponectin gene based on sex, study design and ethnicity showed that there was no significant association between sex, study design and ethnicity and susceptibility to T2D compared to healthy control (Table 3).

**Table 3:** Investigation of association between SNP − 11377 (rs266729) and type 2 diabetes risk using subgroup analyses

Subgroup	Number of studies	OR (95% CI)	Z-value	*P*-value	Test of heterogeneity	
					I^2^ (%)	P
CC genotype	26	0.76 (0.53, 1.09)	− 1.44	0.14	< 0.001	97.7
Ethnicity						
Asian	14	0.96 (0.79, 1.16)	− 0.36	0.71	79.1	< 0.001
European	7	0.78 (0.49, 1.22)	− 1.08	0.27	95.2	< 0.001
American	3	0.25 (0.02, 2.9)	− 1.09	0.27	99.7	< 0.001
African	2	0.83 (0.51, 1.3)	− 0.76	0.44	77.6	0.03
Study design						
Case–control	20	0.69 (0.44, 1.07)	− 1.62	0.10	98.2	< 0.001
Cohort	6	1.04 (0.82, 1.34)	0.38	0.70	72.9	0.002
Sex						
Male	26	0.90 (0.65, 1.23)	0.64	0.52	96	< 0.001
Female	25	1.28 (0.88, 1.86)	1.29	0.19	97.6	< 0.001
CG genotype	26	0.93 (0.72, 1.20)	0.58	0.58	< 0.001	95.3
Ethnicity						
Asian	14	1.05 (0.89, 1.25)	0.64	0.52	71.9	< 0.001
European	7	0.90 (0.66, 1.23)	− 0.62	0.53	89	< 0.001
American	3	0.44 (0.08, 2.24)	− 0.97	0.32	99	< 0.001
African	2	1.2 (0.94, 1.58)	1.51	0.13	34.6	0.21
Study design						
Case–control	20	0.88 (0.64, 1.22)	− 0.72	0.47	96.3	< 0.001
Cohort	6	1.05 (0.93, 1.20)	0.37	0.37	0.00	0.44
Sex						
Male	25	0.99 (0.82, 1.19)	− 0.07	0.94	75.3	< 0.001
Female	24	1.003 (0.81, 1.23)	0.03	0.97	75.8	< 0.001
GG genotype	26	1.008 (0.80, 1.26)	0.067	0.94	< 0.001	75.7
Ethnicity						
Asian	14	1.10 (0.96, 1.27)	1.28	0.14	41.9	0.05
European	7	1.01 (0.75, 1.37)	0.09	0.92	53.6	0.04
American	3	0.50 (0.11, 2.22)	− 0.90	0.36	96.1	< 0.001
African	2	1.41 (0.99, 2.02)	− 0.33	0.05	70	0.06
Study design						
Case–control	20	0.96 (0.73, 1.25)	− 0.28	0.77	80.1	< 0.001
Cohort	6	1.27 (0.98, 1.66)	1.8	0.07	0.00	0.50
Sex						
Male	24	1.10 (0.91, 1.33)	1.05	0.29	0.00	0.50
Female	24	0.90 (0.75, 1.09)	− 1.05	0.45	0.00	0.51

*OR* odds ratio, *I*^*2*^ Higgins index

### Subgroup analysis of + 276 polymorphism

In the + 276 G > T polymorphism with genotypes of TT, GT and GG, subgroup analyses were performed according to the sex, study design and ethnicity. No statistically significant between SNP + 276 (rs1501299) and risk of T2D was found neither in sex (males and females) nor in the study design (case–control and cohort studies). On the other hand, the results of the ethnicity subgroup revealed that + 276 (rs1501299) dominant model (TT *vs.* CG + GG) of European ethnicity relative to other ethnicities could be associated with the risk of T2D. The results of sex subgroup showed no significant association between male and female and the risk of T2D in genotypes reported for + 276 polymorphism of adiponectin gene (Table 4).

**Table 4:** Assessment of association between SNP + 276 (rs1501299) and risk of type 2 diabetes using subgroup analyses

Subgroup	Number of studies	OR (95% CI)	Z-value	*P*-value	Test of heterogeneity	
					I^2^ (%)	P
TT genotype	20	0.87 (0.77, 0.98)	1.84	0.026	38	0.05
Ethnicity						
Asian	9	0.90 (0.75, 1.08)	− 1.07	0.28	0.00	0.56
European	9	0.80 (0.67, 0.97)	− 1.58	0.023	60	0.01
American	2	0.98 (0.72, 1.33)	− 0.29	0.92	60.3	0.11
Study design						
Case–control	14	0.87 (0.70, 1.08)	− 1.22	0.22	52	0.012
Cohort	6	0.80 (0.64, 1)	− 1.93	0.05	0.00	0.71
Sex						
Male	19	0.78 (0.65, 1.03)	− 0.98	0.69	65	0.04
Female	19	0.82 (0.75, 1.21)	− 0.75	0.61	75	0.02
GT genotype	20	0.79 (0.60, 1.02)	− 1.77	0.07	93.8	< 0.001
Ethnicity						
Asian	9	0.99 (0.71, 1.3)	− 0.032	0.97	90.3	< 0.001
European	9	0.67 (0.42, 1.06)	− 1.70	0.08	95.3	< 0.001
American	2	0.61 (0.20, 1.81)	− 0.89	0.37	97.2	< 0.001
Study design						
Case–control	14	0.79 (0.59, 1.05)	− 1.5	0.11	93	< 0.001
Cohort	6	0.79 (0.43, 1.45)	− 0.75	0.45	95.9	< 0.001
Sex						
Male	19	0.76 (0.45, 1.93)	− 1.02	0.76	92	< 0.001
Female	19	0.64 (0.37, 1.78)	− 0.96	0. 45	95	< 0.001
GG genotype	21	0.76 (0.56, 1.04)	-1.6	0.09	96.7	< 0.001
Ethnicity						
Asian	10	1.04 (0.71, 1.24)	0.23	0.81	95.7	< 0.001
European	9	0.57 (0.30, 1.06)	− 1.75	0.08	97.5	< 0.001
American	2	0.58 (0.24, 1.40)	− 1.20	0.22	96.4	< 0.001
Study design						
Case–control	15	0.75 (0.52, 1.06)	− 1.59	0.11	96.3	< 0.001
Cohort	6	0.80 (0.38, 1.68)	− 0.56	0.57	97.7	< 0.001
Sex						
Male	20	0.85 (0.62, 1.75)	− 0.76	0.43	90	< 0.001
Female	20	0.77 (0.34, 1.32)	− 1.61	0.21	85.4	< 0.001

*OR* odds ratio, *I*^*2*^ Higgins index

### Meta-regression analysis

Random-effects meta-regression showed no significant association between genotypes of SNPs of − 11377 with potential confounding factors such as male (slope: 0.0001; 95% CI: − 0.0005 to 0.0007; P = 0.69), female (slope: − 0.0001; 95% CI: − 0.0007 to 0.0004; P = 0.61), age (slope: − 0.43; 95% CI: − 0.82 to 0.91; P = 0.60) and genotyping method (slope: 0.013; 95% CI: − 1.53 to 1.37; *P* = 0.94). On the other hand, for the genotypes of SNP of + 276, no statistical significant association with age and genotyping method was found. While, there was a significant positive association between GT genotype and male individuals (slope: 0.0006; 95% CI: 0.0002 to 0.0009; P < 0.001) in comparison with female individuals (slope: 0.0002; 95% CI: -0.0001 to 0.0005; P = 0.06)]. The analysis of moderator variables showed that they were not the main cause of heterogeneity in the included studies, however sex may modify this association.

## Discussion

This meta-analysis carried out to analyze the correlation between adiponectin (ADIPOQ) gene polymorphisms (SNP − 11377 C > G and SNP + 276 G > T) and risk of T2D among included studies. There are many fundamental mechanisms involved in T2D pathogenesis. However, adiponectin gene polymorphisms have been associated with T2D. In this regard, several studies demonstrated a significant difference in the risk of T2D among individuals with genotypes of adiponectin gene [48, 49]. The adiponectin gene is located on human chromosome 3q27, which is composed of three exons with span 17 kb as a susceptibility locus for T2D [50]. Previous studies have reported conflicting and inconsistent results on the association of ADIPOQ gene − 11377 C > G and + 276 G > T polymorphisms and the risk of T2D. Therefore, we designed this meta-analysis to determine whether these SNPs in the adiponectin gene were correlated with T2D risk in the whole population. In the present meta-analysis, we found a significant association between the TT genotype of + 276 G > T rs1501299 and increased risk of T2D. On the other hand, this meta-analysis demonstrated that the genotypes of (CC, CG and GG) of − 11377 C > G (rs266729) of ADIPOQ gene was no associated with T2D risk.

We performed subgroup analyses according to ethnicity, study design and sex. Subgroup analyses by ethnicity and sex suggested significant association between + 276 G > T polymorphism and T2D risk among male individuals in European population compared to other ethnicities and female individuals. Moreover, we did not find any correlation between T2D risk and ADIPOQ − 11377 C > G polymorphism in the whole population. The obtained results in this study were similar to several previous studies with populations of different ethnicities [24, 51], while, some studies have reported the inconsistent results such that there was no significant association of SNP − 11377 C > G and SNP + 276 G > T with risk of T2D [52–54]. The absence of significant association between genotypes and allele of G and C − 11377 C > G of ADIPOQ gene and risk of T2D might be due to the following results: Differences in the ADIPOQ − 11377 G > C genotype distribution in the ethnic background, location of these SNPs on the ADIPOQ gene, as such SNP − 11377 C > G is located in the ADIPOQ gene promoter region, whereas + 276 G > T is located in the intron 2 region. Thus, studies suggested that genetic variation in the promoter region of this gene with SNP − 11377 could reduce ADIPOQ promoter transcription activity leading to the loss of the relationship between SNP − 11377 and T2D disease. In addition, the ethnicities involved in these two SNPs were different, the majority of populations for − 11377 C > G were Asian, while, those for + 276 G > T were European or Asian.

In order to find the observed heterogeneity, subgroup analyses were performed. Firstly, we analyzed the association between the genotypes (TT, GT, GG and allele C vs. allele G) of + 276 G > T with risk of T2D by subgroup of ethnicity (Asian, European, American and African). The results showed no significant association between genotypes of − 11377 C > G and the risk of T2D, whereas, the TT genotype of + 276 G > C in European population is significantly associated with increased risk of T2D. In the next step, we analyzed this association in subgroups of sex and study design. The pooled results from two SNPs indicated no significant correlation between these SNPs and the risk of T2D in subgroups stratified to sex and study design. For confirming these results, meta-regression was conducted. We found a strong association between susceptibility to T2D and sex in the GT genotype of + 276 G > T. Our meta-analysis data have suggested that males are significantly susceptibility to T2D. This provides some evidence that the association between + 276 (TT and GT) and T2D might be mediated by sex. Therefore, sex may modify this association.

In 2011, Hara et al. have reported that SNP + 276 GG genotype of ADIPOQ gene was associated with T2D in the Japanese population [55]. This result agreed with our meta-analysis data. In contrast to our finding, Hara et al. was found that G vs. C allele of − 11377 C > G rs266729 might be associated with T2D risk. In this regard, there are several explanations for this inconsistency. First, the difference may be owing to the small sample size and the low number of studies in the meta-analysis by Hara et al. relative to our study. Second, the rs266729 polymorphism in the adiponectin gene is not considerable SNP in the whole population. Third, T2D is a complex disease that is affected by environmental agents, life style, socioeconomic condition and individual's susceptibility [56, 57].

Our meta-analysis has several advantages. This study included the most recent published articles on the association between the two SNPs of adiponectin gene and T2D. To enhance the power of strategy search, we used precise inclusion and exclusion criteria and a predefined standard sheet for data extraction. Moreover, to find observed heterogeneity among included studies, we performed subgroup and meta-regression analyses according to the moderator variables of sex, ethnicity, study design and genotyping methods. A comprehensive quality assessment of included studies using NOS checklist detected that the most of the studies had moderate to high quality. In addition, having the high number of studies with large sample size has raised the statistical power of this study.

The limitations of this meta-analysis are that our search was restricted to published studies with English language, which potentially might lead to publication bias. With respect to significant heterogeneity among included studies, the findings of the present study should be interpreted with caution. Finally, the number of studies for subgroup analyses was relatively small, thus, further studies are needed for sex, study design and ethnicity to identify the precise relationship between these two SNPs and increased risk of T2D.

## Conclusion

Regarding to the role of adiponectin level in the control of T2D, this meta-analysis of available studies suggests a strong significant association between the TT genotype of SNP + 276 G > T of adiponectin gene and increased risk of T2D in European population. Sex may modify this association. However, further studies with the higher quality are needed for confirming this association.

## Abbreviations

SNP: Single nucleotide polymorphism
ADIPOQ: Adiponectin gene
T2D: Type 2 diabetes
OR: Odds ratio
CI: Confidence interval
HWE: Hardy–Weinberg equilibrium
NOS: Newcastle Ottawa scale
CMA: Comprehensive Meta-analysis
PRISMA: Preferred reporting items for systematic reviews and meta-analysis

## Declarations

### Availability of data and materials

The data used to support the findings of this study are included in the article. Additional data or information can be requested by contacting the corresponding author.

## References

[1] King H, Aubert RE, Herman WH (1998). Global burden of diabetes, 1995–2025: prevalence, numerical estimates, and projections. Diabetes Care.

[2] Alharbi KK, Abudawood M, Khan IA (2021). Amino-acid amendment of Arginine-325-Tryptophan in rs13266634 genetic polymorphism studies of the SLC30A8 gene with type 2 diabetes-mellitus patients featuring a positive family history in the Saudi population. J King Saud Univ Sci.

[3] Meshkani R (2006). The relationship between homeostasis model assessment and cardiovascular risk factors in Iranian subjects with normal fasting glucose and normal glucose tolerance. Clin Chim Acta.

[4] Cui M (2020). Association between adiponectin gene polymorphism and environmental risk factors of type 2 diabetes mellitus among the Chinese population in Hohhot. BioMed Res Int.

[5] Murea M, Ma L, Freedman BI (2012). Genetic and environmental factors associated with type 2 diabetes and diabetic vascular complications. Rev Diabet Stud RDS.

[6] Mi SQ (2013). BMI, WC, WHtR, VFI and BFI: which indictor is the most efficient screening index on type 2 diabetes in Chinese community population. Biomed Environ Sci.

[7] Mohammadzadeh G (2016). Association of two common single nucleotide polymorphisms (+ 45T/G and+ 276G/T) of ADIPOQ gene with coronary artery disease in type 2 diabetic patients. Iran Biomed J.

[8] Menzaghi C, Trischitta V, Doria A (2007). Genetic influences of adiponectin on insulin resistance, type 2 diabetes, and cardiovascular disease. Diabetes.

[9] Hossain MM, Murali MR, Kamarul T (2017). Genetically modified mesenchymal stem/stromal cells transfected with adiponectin gene can stably secrete adiponectin. Life Sci.

[10] Zhao N (2017). Associations between two common single nucleotide polymorphisms (rs2241766 and rs1501299) of ADIPOQ gene and coronary artery disease in type 2 diabetic patients: a systematic review and meta-analysis. Oncotarget.

[11] Alimi M, Goodarzi MT, Nekoei M (2021). Association of ADIPOQ rs266729 and rs1501299 gene polymorphisms and circulating adiponectin level with the risk of type 2 diabetes in a population of Iran: a case–control study. J Diabetes Metab Disord.

[12] Moher D (2009). Preferred reporting items for systematic reviews and meta-analyses: the PRISMA statement. PLoS Med.

[13] Luchini C (2017). Assessing the quality of studies in meta-analyses: advantages and limitations of the Newcastle Ottawa Scale. World J Meta-Anal.

[14] Begg CB, Mazumdar M (1994). Operating characteristics of a rank correlation test for publication bias. Biometrics.

[15] Egger M (1997). Bias in meta-analysis detected by a simple, graphical test. BMJ.

[16] Borenstein M, Rothstein H. Comprehensive meta-analysis. 1999: Biostat.

[17] Zhang D (2009). A single nucleotide polymorphism alters the sequence of SP1 binding site in the adiponectin promoter region and is associated with diabetic nephropathy among type 1 diabetic patients in the Genetics of Kidneys in Diabetes Study. J Diabetes Complicat.

[18] Hara K (2002). Genetic variation in the gene encoding adiponectin is associated with an increased risk of type 2 diabetes in the Japanese population. Diabetes.

[19] Vasseur F (2002). Single-nucleotide polymorphism haplotypes in the both proximal promoter and exon 3 of the APM1 gene modulate adipocyte-secreted adiponectin hormone levels and contribute to the genetic risk for type 2 diabetes in French Caucasians. Hum Mol Genet.

[20] Gu HF (2004). Single nucleotide polymorphisms in the proximal promoter region of the adiponectin (APM1) gene are associated with type 2 diabetes in Swedish caucasians. Diabetes.

[21] Hu FB (2004). Genetic variation at the adiponectin locus and risk of type 2 diabetes in women. Diabetes.

[22] Gibson F, Froguel P (2004). Genetics of the APM1 locus and its contribution to type 2 diabetes susceptibility in French Caucasians. Diabetes.

[23] Vasseur F (2005). Hypoadiponectinaemia and high risk of type 2 diabetes are associated with adiponectin-encoding (ACDC) gene promoter variants in morbid obesity: evidence for a role of ACDC in diabesity. Diabetologia.

[24] Tso A (2006). Polymorphisms of the gene encoding adiponectin and glycaemic outcome of Chinese subjects with impaired glucose tolerance: a 5-year follow-up study. Diabetologia.

[25] Schwarz P (2006). Haplotypes in the promoter region of the ADIPOQ gene are associated with increased diabetes risk in a German Caucasian population. Horm Metab Res.

[26] Gable D (2007). Common adiponectin gene variants show different effects on risk of cardiovascular disease and type 2 diabetes in European subjects. Ann Hum Genet.

[27] Olckers A (2007). Protective effect against type 2 diabetes mellitus identified within the ACDC gene in a black South African diabetic cohort. Metabolism.

[28] Sun H (2008). The association of adiponectin allele 45T/G and − 11377C/G polymorphisms with Type 2 diabetes and rosiglitazone response in Chinese patients. Br J Clin Pharmacol.

[29] Hivert M-F (2008). Common variants in the adiponectin gene (ADIPOQ) associated with plasma adiponectin levels, type 2 diabetes, and diabetes-related quantitative traits: the Framingham Offspring Study. Diabetes.

[30] Wang X (2009). APM1 gene variants− 11377C/G and 4545G/C are associated respectively with obesity and with non-obesity in Chinese type 2 diabetes. Diabetes Res Clin Pract.

[31] Min Y (2008). Identification of a regulatory single nucleotide polymorphism in the adiponectin (APM1) gene associated with type 2 diabetes in Han nationality. Biomed Environ Sci.

[32] Wang Y (2009). Association study of the single nucleotide polymorphisms in adiponectin-associated genes with type 2 diabetes in Han Chinese. J Genet Genomics.

[33] Chiodini BD (2010). Adiponectin gene polymorphisms and their effect on the risk of myocardial infarction and type 2 diabetes: an association study in an Italian population. Ther Adv Cardiovasc Dis.

[34] Karimi H (2019). The impact of adiponectin gene polymorphisms on the insulin resistance index in patients with diabetes and newly diagnosed type 2 diabetes. Int J Diabetes Metab.

[35] Palit SP (2020). A genetic analysis identifies a haplotype at adiponectin locus: association with obesity and type 2 diabetes. Sci Rep.

[36] Ramya K (2013). Genetic association of ADIPOQ gene variants with type 2 diabetes, obesity and serum adiponectin levels in south Indian population. Gene.

[37] Mtiraoui N (2012). Single-nucleotide polymorphisms and haplotypes in the adiponectin gene contribute to the genetic risk for type 2 diabetes in Tunisian Arabs. Diabetes Res Clin Pract.

[38] Nomani H (2019). Association between the − 11377 C/G and − 11391 G/A polymorphisms of adiponectin gene and adiponectin levels with susceptibility to type 1 and type 2 diabetes mellitus in population from the west of Iran, correlation with lipid profile. J Cell Biochem.

[39] Populaire C (2003). Does the − 11377 promoter variant of APM1 gene contribute to the genetic risk for type 2 diabetes mellitus in Japanese families?. Diabetologia.

[40] Menzaghi C (2002). A haplotype at the adiponectin locus is associated with obesity and other features of the insulin resistance syndrome. Diabetes.

[41] Fumeron F (2004). Adiponectin gene polymorphisms and adiponectin levels are independently associated with the development of hyperglycemia during a 3-year period: the epidemiologic data on the insulin resistance syndrome prospective study. Diabetes.

[42] De Courten BV (2005). Common polymorphisms in the adiponectin gene ACDC are not associated with diabetes in Pima Indians. Diabetes.

[43] Lee Y (2005). Genetic association study of adiponectin polymorphisms with risk of Type 2 diabetes mellitus in Korean population. Diabet Med.

[44] González-Sánchez JL (2005). An SNP in the adiponectin gene is associated with decreased serum adiponectin levels and risk for impaired glucose tolerance. Obes Res.

[45] Yang W-S (2007). Adiponectin SNP276 is associated with obesity, the metabolic syndrome, and diabetes in the elderly. Am J Clin Nutr.

[46] Szopa M (2009). Variants of the adiponectin gene and type 2 diabetes in a Polish population. Acta Diabetol.

[47] Mohammadzadeh G, Zarghami N (2009). Associations between single-nucleotide polymorphisms of the adiponectin gene, serum adiponectin levels and increased risk of type 2 diabetes mellitus in Iranian obese individuals. Scand J Clin Lab Invest.

[48] Vionnet N (2000). Genomewide search for type 2 diabetes–susceptibility genes in French Whites: evidence for a novel susceptibility locus for early-onset diabetes on chromosome 3q27-qter and independent replication of a type 2–diabetes locus on chromosome 1q21–q24. Am J Human Genet.

[49] Mori Y (2002). Genome-wide search for type 2 diabetes in Japanese affected sib-pairs confirms susceptibility genes on 3q, 15q, and 20q and identifies two new candidate Loci on 7p and 11p. Diabetes.

[50] Kissebah AH (2000). Quantitative trait loci on chromosomes 3 and 17 influence phenotypes of the metabolic syndrome. Proc Natl Acad Sci.

[51] Ru Y (2005). Association of SNP276 in adiponectin gene with type 2 diabetes mellitus and insulin sensitivity. Zhonghua Yi Xue Yi Chuan Xue Za Zhi.

[52] Wang S-F (2007). The correlation between adiponectin gene polymorphism and type 2 diabetes. Chin J Endocrinol Metab.

[53] Xia H et al. Correlation between single nucleotide polymorphism of adiponectin gene and type 2 diabetes in Chinese. Chin J Endocrinol Metab. 1986(03).

[54] Dong Y et al. Relationship between adiponectin gene polymorphisms and type 2 diabetes. Acad J Shanghai Second Med Univ. 2004;12(008).

[55] Han L (2011). Associations between single-nucleotide polymorphisms (+ 45T> G,+ 276G> T,− 11377C> G,− 11391G> A) of adiponectin gene and type 2 diabetes mellitus: a systematic review and meta-analysis. Diabetologia.

[56] Nair M (2007). Diabetes mellitus, part 1: physiology and complications. Br J Nurs.

[57] Liao N (2013). Association between the ghrelin Leu72Met polymorphism and type 2 diabetes risk: a meta-analysis. Gene.

